# Aggressive Treatment of Patients with Metastatic Colorectal Cancer Increases Survival: A Scandinavian Single-Center Experience

**DOI:** 10.1155/2013/727095

**Published:** 2013-06-06

**Authors:** Kristoffer Watten Brudvik, Simer Jit Bains, Lars Thomas Seeberg, Knut Jørgen Labori, Anne Waage, Kjetil Taskén, Einar Martin Aandahl, Bjørn Atle Bjørnbeth

**Affiliations:** ^1^Centre for Molecular Medicine Norway, University of Oslo, 0318 Oslo, Norway; ^2^Biotechnology Centre, University of Oslo, 0317 Oslo, Norway; ^3^Department of Hepato-Pancreato-Biliary Surgery, Oslo University Hospital, 0424 Oslo, Norway; ^4^Department of Infectious Diseases, Oslo University Hospital, 0424 Oslo, Norway; ^5^Department of Transplantation Surgery, Oslo University Hospital, 0424 Oslo, Norway

## Abstract

*Background*. We examined overall and disease-free survivals in a cohort of patients subjected to resection of liver metastasis from colorectal cancer (CRLM) in a 10-year period when new treatment strategies were implemented. *Methods*. Data from 239 consecutive patients selected for liver resection of CRLM during the period from 2002 to 2011 at a single center were used to estimate overall and disease-free survival. The results were assessed against new treatment strategies and established risk factors. *Results*. The 5-year cumulative overall and disease-free survivals were 46 and 24%. The overall survival was the same after reresection, independently of the number of prior resections and irrespectively of the location of the recurrent disease. The time intervals between each recurrence were similar (11 ± 1 months). Patients with high tumor load given neoadjuvant chemotherapy had comparable survival to those with less extensive disease without neoadjuvant chemotherapy. Positive resection margin or resectable extrahepatic disease did not affect overall survival. *Conclusion*. Our data support that one still, and perhaps to an even greater extent, should seek an aggressive therapeutic strategy to achieve resectable status for recurrent hepatic and extrahepatic metastases. The data should be viewed in the context of recent advances in the understanding of cancer biology and the metastatic process.

## 1. Introduction

The incidence of colorectal cancer (CRC) is increasing and is now the fourth leading cause of cancer deaths worldwide [1]. Twenty percent of the patients present with synchronous liver metastases and another 30–40% develop liver metastases during followup [2]. Hepatic resection remains the only potentially curable treatment and is now offered to 20–25% of the patients whereas only 10% were selected for this treatment ten years ago [3]. The main exclusion criteria for liver resection of colorectal liver metastases (CRLMs) are nonresectable liver metastasis (tumor growth into both portal branches and/or into both left and right liver vein), inadequately functioning residual liver parenchyma, or nonresectable extrahepatic disease. These exclusion criteria have all been challenged in recent years. Close followup after primary CRC (early detection of metastasis), implementation of new surgical techniques including two-stage hepatectomy with portal vein embolization [4, 5] and transplantation methods, and the introduction of new chemotherapy and biological agents capable of converting inoperable cases to a resectable status by tumor downsizing have increased the number of patients eligible for resection of liver metastases [6, 7]. As a consequence, reresection of patients with recurrent disease is now offered to an increasing number of selected patients [8, 9].

A cohort of 239 patients with CRC and synchronous or metachronous CRLMs eligible for liver resection with curative intent was followed from 2002 to 2011. The aim of the study was to examine overall and disease-free survivals related to number of resections, therapeutic downsizing, surgical technique, and other factors considered to have prognostic value.

## 2. Patients and Methods

### 2.1. Patients Selection and Management

All patients were considered preoperatively by a multidisciplinary team. The assessment included computed tomography (CT) of the abdomen and chest with the addition of magnetic resonance (MR) or ultrasonography with contrast when resectability could not be determined after CT. Positron emission tomography (PET) became available in 2009 and was used to assess extrahepatic disease in selected cases. Intraoperative contrast-enhanced ultrasonography (CEU) was used in every procedure after 2007 to assess resectability and tumor expansion as previously described [10]. Preoperative carcinoembryonic antigen (CEA) levels were determined in all patients in the most recent 5-year period. 

The Brisbane terminology was applied to classify the liver resections [11]. Laparoscopic resection was introduced during the last 4-year period for selected patients with small, subcapsular lesions or lesions in the lateral or lower segments (segments II, III, IVb, V, and VI). The term two-stage hepatectomy was used where the first surgical step included nonanatomical resection on one side combined with postoperative portal vein embolization of the most affected side, followed by a second step with formal resection of the side with remaining disease.

### 2.2. Data Collection and Statistics

Information was retrieved from medical records, including operation, radiology, and pathology reports. Followup was performed in our outpatient clinic at 4, 8, and 12 months and from the second postoperative year at 6-month intervals for a total of 5 years. Size of the largest tumor and number of metastases were used to compare tumor load between subgroups and the tumor load was calculated by points based on the worst score for each parameter in the Basingstoke Predictive Index (8 points if diameter of the largest tumor >10 cm; adapted to 8 points/cm and 4 points if >3 metastases; adapted to 1 point/number of metastases and multiplied by 10 to produce a score where size and load are equally representative). We assessed our results against established risk factors reported by others [12–14] (see supplemental Figure S1 in Supplementary Material available online at http://dx.doi.org/10.1155/2013/727095).

Filemaker Pro 9.0 (Santa Clara, CA, USA) was used to register data that were analyzed in SPPS 16.0 (Chicago, IL, USA). Graphs were made in SigmaPlot 11.0 (San Jose, CA, USA). Kaplan-Meier plots and log-rank (Mantel-Cox) comparisons were used to compute cumulative survival data. Pearson's chi-square test was used to compare ratios. Group means were compared using Student's *t*-test if the variables passed a normality test; otherwise medians where compared with rank-sum test. The study and database were approved by the Oslo University Hospital Data Protection Officer for Research.

## 3. Results

Patients undergoing surgery for CRLMs (adenocarcinoma) were registered partly prospectively and partly retrospectively in a database from October 2002 to August 2011. In the study period, a total of 268 patients were initially included. Of these, 27 patients were deemed inoperable intraoperatively due to extensive hepatic or extrahepatic metastatic disease and excluded from the study. Five of these patients were scheduled for two-stage hepatectomy [15] but were inoperable at the 2nd surgical step. In addition, two patients in the original database received liver transplants [16] and were excluded resulting in a total of 239 patients analyzed in the present study.

The cohort of 239 patients with metastatic colorectal cancer patients resected for liver metastasis was examined for overall and disease-free survivals. Characteristics of the patient cohort and primary tumor are presented in supplemental Table S1.

### 3.1. Liver Resection

Liver resection of the CRLMs was successfully accomplished in 90.7% (*n* = 214) of the planned single-stage procedures and in 83.3% (*n* = 25) of the planned two-stage procedures (mean age 64.3 years, range 26–89 years; 118 females; Table 1). A total of 353 surgical procedures were registered in the cohort, representing primary resections (*n* = 239), secondary resections (*n* = 65), and tertiary resections (*n* = 21); 2nd step of two-stage hepatectomy (*n* = 25) and 2nd step of single-stage surgery converted to two-stage without embolization (*n* = 3; see supplemental Table S2 for details of the surgical procedures). In 16 patients with rectum cancer (after 2008), a liver-first approach was chosen [17]. The type of resection and details are presented in supplemental Table S2.

### 3.2. Overall and Oncologic Outcome

**Figure 1:**
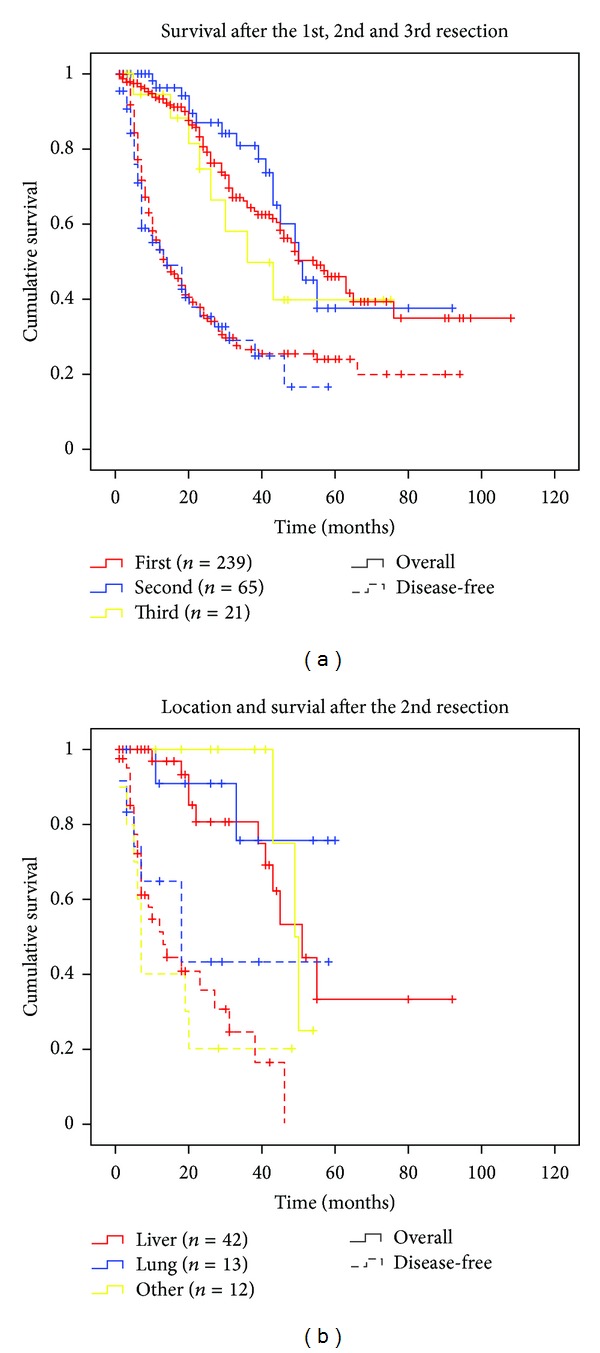
Overall and disease-free survivals of colorectal cancer patients following liver resection of primary or recurring metastasis. (a) The Kaplan-Meier plots showing overall (full line) and disease-free (dashed line) survivals in the total population and after the first, second, and third resections. +: censored cases. (b) Survival after a second resection of colorectal metastases at different locations (67 procedures in 65 patients; one patient resected for liver and lung; one patient resected for liver and lymph recurrent disease), data presented as in (a).

Intraoperative mortality was zero; however three (1.3%) died within 30 days after the surgical procedure (day 17, 29, and 29, resp.). At a median observation time of 24 months (range 1–108 months by October 2011) 99 of the 239 patients (41.1%) were alive and disease-free, and 64 (26.8%) of the patients were alive with recurrent disease and were currently receiving palliative treatment or were undergoing evaluation for re-resection. Furthermore, 66 of the patients (27.6%) had died of the disease and 10 (4.2%) had died of unrelated reasons or of unknown cause. The cumulative overall 5- and 9-year survivals were 46.0 and 34.9%, respectively, and comparable to that of other centers (36 to 58% and 23 to 36% for 5- and 10-year survivals, respectively [12, 13, 18–23]). The disease-free survivals were 24.0 and 20.0% for 5- and 7-year follow-up periods, respectively (Figure 1(a)). The locations of recurrent disease are presented in supplemental Table S3 and show a shift in target organ as the disease continues to recur.

### 3.3. Resection of Recurring Metastases

In the observation period 146 patients presented with a second recurrent disease and surgery with curative intent was performed in 65 (44.8%) of them. A third recurrent disease presented in 69 patients and surgery with curative intent was performed in 21 (30.4%). Overall survival appeared to be the same after the first, second, and third resections and the disease-free survival was similar in the groups resected once and twice (Figure 1(a)). Furthermore, survival after the second resection was comparable independently of whether the location of the recurrent disease was to the liver, lung, or elsewhere (Figure 1(b) and supplement Table S3). The average time from surgery of the primary tumor to resection of the first CRLM was 11.7 months; the average time from surgery for the CRLM to the presentation of a second recurrent disease (irrespective of localization) was 10.1 whereas the mean time from the second to the third recurrence was 11.0 months.

### 3.4. Downsizing and Neoadjuvant Chemotherapy

**Figure 2:**
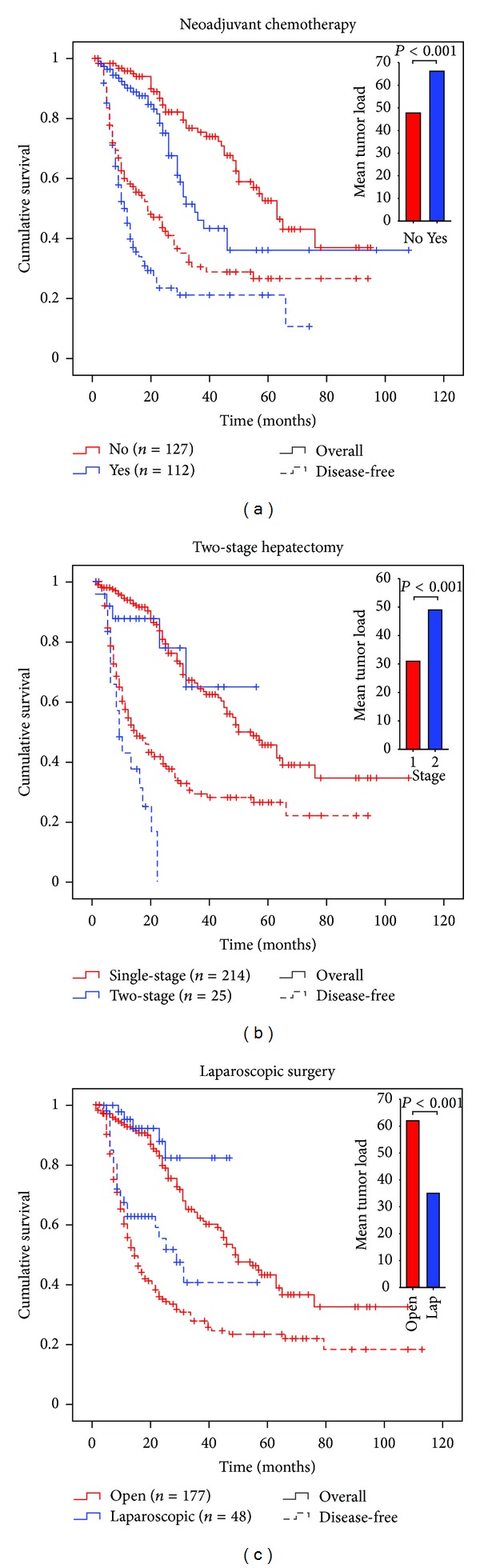
Overall and disease-free survivals after neoadjuvant chemotherapy and the first resection for colorectal metastases (a), after two-stage hepatectomy with portal vein embolization (b), or after laparoscopic surgery (c). Data presented as in Figure 1(a). Bar charts (top left corners) show mean *tumor load score* calculated as indicated in the Patients and Methods section. Independent *t*-test was used to compare mean tumor load in subgroups.

Preoperative (neoadjuvant) chemotherapy was given to 46.9% of the patients (*n* = 112, Table 1). The indications for neoadjuvant chemotherapy changed during the study period. In the first 5-year period (2002–2006, *n* = 24; 32.9%), neoadjuvant therapy was primarily given to nonresectable patients. In the last 5-year period (2007–2011, *n* = 88; 53.0%), the indications were broader and included patients with high tumor load (3 or more metastases *or* large metastasis above 30 mm (diameter) *or* synchronous metastases) and from January 2010 young patients *with* elevated CEA *and* ECOG performance status 0-1 [24]. The cumulative overall 5-year survival of patients receiving neoadjuvant chemotherapy was 36.1% versus 52.6% in the nonneoadjuvant group (*P* = 0.008) whereas survival between the two groups appeared similar during past 80 months (Figure 2(a)). The 5-year disease-free survival was 21.0% in the neoadjuvant group versus 26.5% in the non-neoadjuvant group (*P* = 0.025). Tumor load was significantly higher in the neoadjuvant group (Figure 2(a), insert).

Stratification of neoadjuvant chemotherapy combined with tumor load revealed that the survival in patients with high tumor load receiving preoperative chemotherapy was increased compared to that of patients not receiving chemotherapy. In contrast, patients with low tumor load who did not receive neoadjuvant chemotherapy had increased survival compared to the survival of those who received chemotherapy.

### 3.5. Two-Stage Hepatectomy

Thirty patients received two-stage hepatectomy from 2008 due to bilateral disease. Five of them (16.7%) were inoperable at the second surgical step and excluded from the study and results. Patients selected for two-stage hepatectomy had a higher risk of developing recurrence as all patients in this group presented with recurrent disease within 2 years compared to a 60.7% recurrence in patients subject to a single-stage procedure in the same time interval (Figure 2(b)). Nonetheless, the overall survival in the group that received two-stage hepatectomy appeared comparable to that of the single-stage procedure group with the limited data available. Of the 25 patients who underwent the two-stage procedure, six (24.0%) were alive with a mean observation time of 17 months (range 6–45) and were reported to be disease-free at the time of examination of the cohort.

### 3.6. Laparoscopic Surgery

Since the introduction of laparoscopic resection in 2008, 60 patients have been selected for this procedure of which 48 were successfully completed (20.0% conversion rate). In addition, the first step of planned two-stage surgery was performed laparoscopically in two patients. Patients selected for a laparoscopic approach had a better outcome than patients selected for open surgery (Figure 2(c)) and lower tumor load (insert).

### 3.7. Prognostication of Colorectal Liver Metastasis

**Figure 3:**
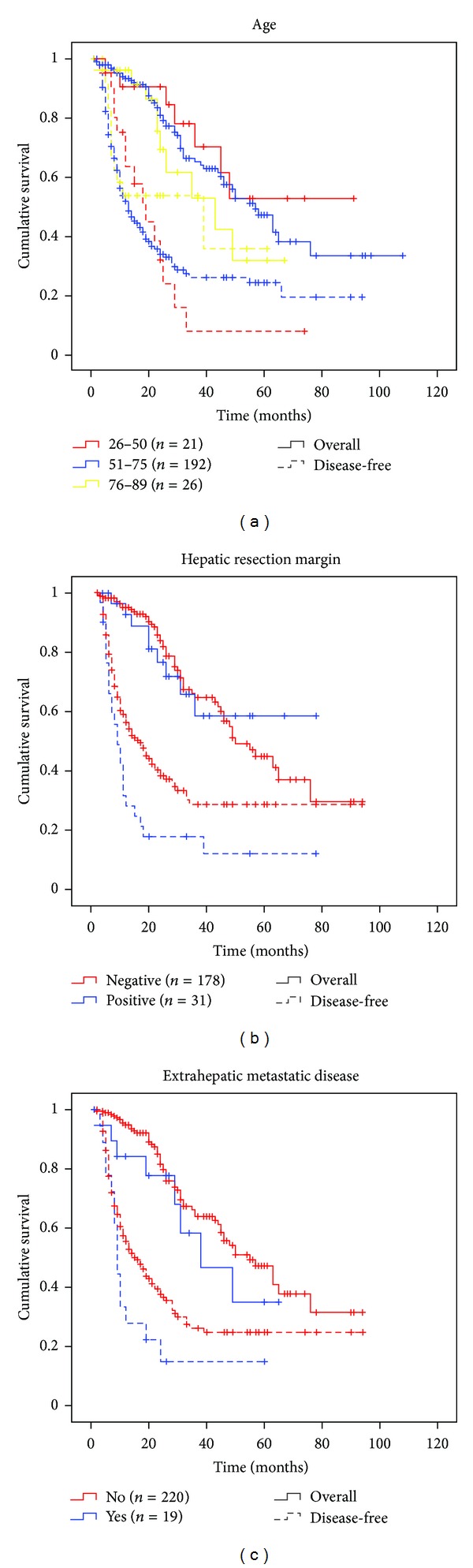
Overall and disease-free survivals stratified on risk factors. Data presented by the Kaplan-Meier plots with overall (solid line) and disease-free (dashed line). Age group (a), hepatic resection margin (b), and extrahepatic disease at the time point of surgery, as indicated.

Primary tumor lymph node status, histological differentiation grade, synchronous or metachronous disease, tumor size (metastasis), numbers of metastases, affected liver segments, and CEA levels turned out to be prognostic markers affecting survival (supplemental Figure S1). Furthermore, we found ascending age to be a positive prognostic marker for disease-free survival (Figure 3(a)). In contrast, examining the overall survival, we observed an apparent higher mortality in the older patient group. Extrahepatic disease (*n* = 19, Figure 3(c)) and positive hepatic resection margin (R1/R2, *n* = 31, Figure 3(b)) impaired disease-free survival. However, overall survival was not affected by resectable extrahepatic disease or positive resection margins.

In the present material, men had better overall outcome and rectum cancer correlated positively with overall and disease-free survivals (supplemental Figure S1a and b).

## 4. Discussion

Surgical treatment of CRLMs is offered to an increasing number of patients with metastatic CRC [3]. This has opened several new avenues in the treatment of this patient group, and as a consequence fundamental questions in tumor biology and clinical strategies are now being challenged. Recent reports on survival following re-resection of CRLMs and resection of extrahepatic metastases support a more aggressive treatment practice [8, 25–27]. Neoadjuvant chemotherapy to downsize CRLMs increases the number of resectable cases and provides the opportunity to target a larger patient population. In the present study patients who received neoadjuvant chemotherapy to downsize CRLMs reached a long-term overall survival comparable to that of primary resectable patients, despite widespread disease. It is, in this connection, important to note that tumor load calculations were performed based on information in the pathology reports and are therefore postneoadjuvant chemotherapy which means that they underreport the initial tumor load.

 Here we report that second and third resections of recurring CRLMs should be considered when possible and that resection also should be assessed in patients with extrahepatic recurrences as their prognosis does not appear to be worse, but for strict recommendations randomized clinical trials would be needed. Patients selected for laparoscopic approach had a better outcome than patients selected for open surgery (Figure 2(c)), which may be related to the selection criteria and tumor load. A recent report indicates laparoscopic results comparable to those of open surgery when the selection criteria are identical for the two procedures [28]. Females are reported to have a better prognosis after liver resection for CRLMs than males and colon cancer to have better prognosis than rectum cancer [14]. However, in the present material, men had better overall outcome and a primary rectum cancer correlated positively with overall and disease-free survivals (supplemental Figure S1a and b). The latter may reflect observations that neoadjuvant radiochemotherapy and a more radical surgical technique, total mesorectal excision (TME), have improved survival after treatment for rectum cancer. Ascending age has been reported to be both negatively and positively correlated with survival [12, 14, 29]. We found ascending age to be a positive prognostic marker for disease-free survival (Figure 3(a)), which may be related to more aggressive tumor biology in younger patients. A higher proportion of the young patients presented with recurrent disease and the recurrence occurred more rapidly than in older patients. Interestingly, the overall survival curves were inverted compared to disease-free survival with respect to the different age groups (Figure 3(a)). This could be explained by increased surgical and adjuvant efforts towards young and otherwise healthy patients. If this is the case, this in itself could be proof that an aggressive approach could produce long-term survivors.

Recent genetic and molecular studies of metastatic malignant disease indicate that metastases often develop in parallel to the primary tumor from an early stage, and that the tumor biology of the metastases is not necessarily more aggressive than that of the primary tumor [30, 31]. Previous studies addressing the growth rate of various malignant tumors including CRC indicate that the tumor volume doubling time (TVDT) of the metastases is comparable to that of the primary tumor [32]. This may suggest that metastases identified late and removed in re-resection procedures could represent tumors that were not recognized at the time of surgery of the primary tumor or the first metastasis due to their small size, rather than progressively developing and increasingly aggressive metastases. In our cohort, this may be reflected in the observation that overall survival and oncologic outcome were comparable in patients with successful outcome of the first resection and those who required a second or third resection. In line with this thinking, “recurrent disease” may be a misnomer as the disease may not be recurring but continues to deliver earlier established metastases growing in parallel and reaching a size that allows diagnosis at different time points following the primary surgery. Thus, resection of metastases may in many cases represent incremental tumor-reductive surgery rather than treatment of recurrent disease. This may also help to explain why the discrimination between R0 and R1/R2 resections of CRC metastases is not crucial with respect to overall survival (our data and [33]). These findings are in contrast to expected results from current scoring systems [12, 14]. In conclusion, metastatic disease is systemic or multifocal in its nature and may encompass unrecognized foci at the time of surgery in most if not all patients irrespectively of the presentation at diagnosis. Eradication of all tumor tissue may therefore not be conceivable in the majority of the patients. However, this recognition should not preclude an aggressive treatment approach with repeated resections that continue to reduce tumor load. 

Extrahepatic metastatic spread has previously been considered an end-stage disease. In recent years, however, combined liver and lung resections have produced long-time survivors. Consequently, pulmonary metastasis alone is no longer considered an exclusion criterion for surgery [34]. Six patients in our cohort presented with concomitant pulmonary lesions with uncertain malignant potential; resection of CRLMs was performed with a “wait and watch” approach taken with respect to the development of pulmonary metastases. In four patients the pulmonary metastases progressed but were accessible by lung resection and three of these were alive and had remained disease-free until the time of examination of the cohort. It is also interesting to observe that patients resected for recurrent disease to the lung after first having performed hepatic resection had comparable and maybe even better survival compared to those repeatedly resected for recurrent disease in the liver, and as such, pulmonary metastases may not be a sign of an explosive metastatic spread [35].

## 5. Conclusion

The presented results indicate that surgical treatment, when possible with or without neoadjuvant treatment, significantly prolongs life and may in some cases cure the disease, even when extensive. Hence, efforts to identify or induce technically resectable cases are crucial and include early detection of metastatic spread, improved surgical techniques, and neoadjuvant chemotherapy to downsize the metastases. Patients that become resectable after neoadjuvant therapy appear to reach the same survival rate as that of as patients that are primary resectable. Furthermore, neoadjuvant therapy may also be used for selection of patients with the best prognosis after surgical resection, as patients that progress during ongoing treatment will probably not benefit from surgical treatment. The present report adds to the current knowledge base of the outcome of an aggressive treatment approach to metastatic CRC. Although metastatic CRC has a poor prognosis, surgical treatment has clear patient benefit and strategies to make patients resectable and available for surgery should be pursued.

## Supplementary Material

Supplemental Table S1 Characteristics primary tumor
Supplemental Table S2 Type of resections, the first liver resection in 239 patients. *∗*Alone or in combination with formal liver resection. *∗∗*Always in combination with formal liver resection or non-anatomic resection
Supplemental Table S3 Location (Loc) and surgery (Rec) of recurrent disease, Number of patients (% of group). Some patients presented with and were treated for recurrent disease in more than one location at the same time
Supplemental Table S4 Chemotherapy to liver metastases, Number of patients. FLOX - fluorouracil (5-FU) with leucovorin and oxaliplatin, FLIRI - fluorouracil (5-FU) with leucovorin and irinotecan, FLV - fluorouracil (5-FU) with leucovorin. *∗*Avastin is administered in combination with any of the above Figure Legends
Supplemental Figure S1 Kaplan-Meier plots showing overall (full line) and disease-free (dashed line) survival in patients after liver resection for colorectal metastatic disease. +: Censored cased. Subgroups presented are based on selected risk factors from the Basingstoke Predictive Index (BPI) and the Fong Predictive Nomogram

## Figures and Tables

**Table 1:** Characteristics of liver metastases.

	Value
Age (range)	64.3 (26–89)
Female/male	118/121
BPI score (range) (*n* = 94)	5.98 (0–27)
Metachronous/synchronous metastases	133/106
Number of tumors in the liver	
1/2/3/4/5/6 or more	87/52/31/14/12/18
Missing	25
Size diameter mm (mean/median/min/max)	29/22/2/155
Missing	15
Number of segments involved	
1/2/3/4/5/6 or more	63/70/40/29/13/6
Missing	18
CEA (*n* = 140)	55.5 (0.7–1400.0)
Extra hepatic disease	19
Neoadjuvant chemotherapy	
Yes/no/na	112/127/0
Adjuvant chemotherapy	
Yes/no/na	128/89/22
