# Supplementary material for: Aggressive Treatment of Patients with Metastatic Colorectal Cancer Increases Survival: A Scandinavian Single-Center Experience
Source: HPB Surg. 2013 Jun 6;2013:727095. doi: 10.1155/2013/727095 (PMC3690245; doi:10.1155/2013/727095)
Supplement: Supplementary file 1 — Supplemental Table S1 Characteristics primary tumor Supplemental Table S2 Type of resections, the first liver resection in 239 patients. ∗Alone or in combination with formal liver resection. ∗∗Always in combination with formal liver resection or non-anatomic resection Supplemental Table S3 Location (Loc) and surgery (Rec) of recurrent disease, Number of patients (% of group). Some patients presented with and were treated for recurrent disease in more than one location at the same time Supplemental Table S4 Chemotherapy to liver metastases, Number of patients. FLOX - fluorouracil (5-FU) with leucovorin and oxaliplatin, FLIRI - fluorouracil (5-FU) with leucovorin and irinotecan, FLV - fluorouracil (5-FU) with leucovorin. ∗Avastin is administered in combination with any of the above Figure Legends Supplemental Figure S1 Kaplan-Meier plots showing overall (full line) and disease-free (dashed line) survival in patients after liver resection for colorectal metastatic disease. +: Censored cased. Subgroups presented are based on selected risk factors from the Basingstoke Predictive Index (BPI) and the Fong Predictive Nomogram [file 727095.f1.pdf]

SUPPLEMENTARY INFORMATION

To

Brudvik et al. *Aggressive treatment of patients with metastatic colorectal cancer increases survival*

Legends to Tables

Supplemental Table S1 Characteristics primary tumor

Supplemental Table S2 Type of resections

The first liver resection in 239 patients. *Alone or in combination with formal liver resection. **Always in combination with formal liver resection or non-anatomic resection

Supplemental Table S3 Location (Loc) and surgery (Rec) of recurrent disease

Number of patients (% of group). Some patients presented with and were treated for recurrent disease in more than one location at the same time

Supplemental Table S4 Chemotherapy to liver metastases

Number of patients. FLOX - fluorouracil (5-FU) with leucovorin and oxaliplatin, FLIRI - fluorouracil (5-FU) with leucovorin and irinotecan, FLV - fluorouracil (5-FU) with leucovorin. *Avastin is administered in combination with any of the above

Figure Legends

Supplemental Figure S1 Kaplan-Meier plots showing overall (full line) and disease-free (dashed line) survival in patients after liver resection for colorectal metastatic disease. +: Censored cased. Subgroups presented are based on selected risk factors from the Basingstoke Predictive Index (BPI) and the Fong Predictive Nomogram

Supplemental Table S1 Characteristics primary tumour

	Value
Age (range)	63.4 (26-85)
Primary tumour location	
Colon / Rectum / Na	144 / 88 / 7
Primary tumour Ln status	
Neg / Pos / Na	81 / 116 / 42
Primary tumour Diff status	
Well / Moderate / Poor / Na	21/ 142 / 15 / 61
Primary surgery in referral hospital	184
Referral hospitals	12

Supplemental Table S2 Type of liver resections

	No. patients
Open / Laparoscopic / Converted / Two-stage	177 / 48 / 12 / 2
Two-stage hepatectomy	25
Anatomic segmentectomy	36
Right side hemihepatectomy	70
Left side hemihepatectomy	16
Left lateral sectionectomy	18
Non-anatomic resections*	161
Radiofrequency ablation**	15
Surgeons	
#1 / #2 / #3 / #4 / #5 / #6 / Others	86 / 60 / 40 / 14 / 10 / 6 / 23
Free Margin	
Yes / No / Na	178 / 30 / 31
Adenocarcinoma	239
Rec2 re-resections	65
Rec3 re-resections	21

Supplemental Table S3 Location (Loc) and surgery (Rec) of recurrent disease

	Loc2 (n=146)	Rec2 (n=65)	Loc3 (n=69)	Rec3 (n=21)
Liver	87 (59.6%)	42 (64.6%)	28 (40.6%)	10 (47.6%)
Lung	50 (34.2%)	13 (20.0%)	24 (34.8%)	9 (42.9%)
Lymph	14 (9.6%)	5 (7.7%)	12 (17.4%)	1 (4.8%)
Peritoneum	10 (6.8%)	2 (3.1%)	0	0
Local	6 (4.1%)	2 (3.1%)	3 (4.3%)	1 (4.8%)
Skin	5 (3.4%)	1 (1.5%)	4 (5.8%)	1 (4.8%)
Thoracic wall	1 (0.7%)	1 (1.5%)	2 (2.9%)	1 (4.8%)
Pelvic organs	1 (0.7%)	1 (1.5%)	4 (5.8%)	2 (9.5%)
Bone	1 (0.7%)	0	4 (5.8%)	0
Pancreas	0	0	1 (1.4%)	0
Brain/Meninges	0	0	5 (7.2%)	1 (4.8%)

Supplemental Table S4 Chemotherapy to liver metastases

	Neoadjuvant	Adjuvant
FLOX	86	98
FLIRI	19	18
FLV	7	12
Avastin*	16	20

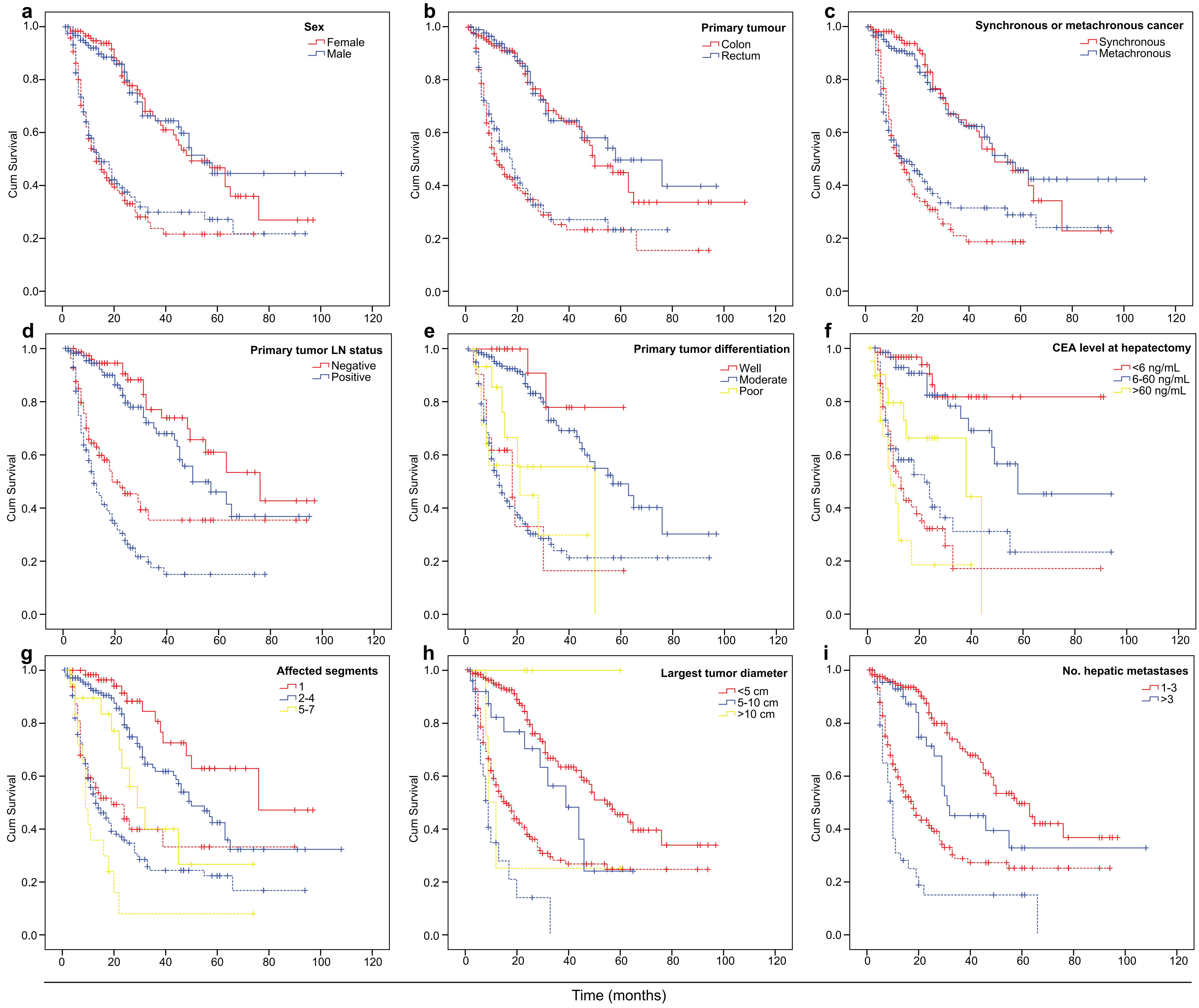

Supplemental Figure S1. Brudvik et al.
